# Chromium propionate enhanced the production performance of yellow-feathered broilers under chronic heat stress exposure by improving intestinal health

**DOI:** 10.1007/s44154-026-00320-6

**Published:** 2026-06-22

**Authors:** Jinglong Chen, Chuangchuang Lei, Xuming Guo, Dan Shao, Changzheng Guo, Shourong Shi

**Affiliations:** 1 Jiangsu Institute of Poultry Science, Yangzhou, 225125 China; 2 Huazhong Agricultural University, Wuhan, 430070 China

**Keywords:** Yellow-feathered broilers, Heat stress, Chromium propionate, Growth performance, Mucosal barrier, Gut microbiota

## Abstract

Heat stress is one of the key factors affecting broiler production. This study aimed to investigate the protective effects and underlying mechanisms of chromium propionate (CrP) against heat stress in broilers. A total of 180 yellow-feathered broilers were randomly divided into 3 groups, with 6 replicates per group and 10 broilers per replicate. The CON and HS groups were fed with a basic diet, while the PAC group was supplemented with 0.2 mg/kg CrP. Following pre-feeding for 7 days, HS and PAC groups were exposed to cyclic HS (8 h at 30, 32, and 34 °C, respectively) for two weeks, while the control group was maintained at 26 ℃ ± 1 ℃. The experiment was designed in accordance with a randomized block design, and data were analyzed by one-way ANOVA. The results demonstrated that dietary supplementation with CrP eased heat stress–induced reductions in BW and ADG as well as increased the feed conversion ratio. Heat stress considerably decreased gut length and V/C across all intestinal segments. Moreover, CrP suppressed heat stress–induced increases in serum corticosterone and lipopolysaccharide levels while enhancing intestinal glutathione peroxidase activity. Gene expression analysis of jejunal mucosa revealed that CrP downregulated heat stress-induced upregulation of heat shock proteins and inflammation-related genes *COX2* and *NLRP3* while enhancing the expression of *Mucin2*, *Occludin* and *ZO-1*. Analysis of 16S rRNA sequencing revealed distinct differences in microbial composition and potential functional shifts among groups. At the genus level, heat stress decreased *Alistipes* relative abundance along with marked increases in *Lactobacillus* count; conversely, CrP reversed these trends. LEfSe identified *Lactobacillus* and *Parabacteroides* as the predominant bacteria in the HS group, while *Gemmatimonas* and *Cetobacterium* predominated the PAC group. Functional prediction analyses indicated that heat stress suppressed carbohydrate and amino acid metabolism while enhancing RNA degradation pathways; these effects were effectively reversed by CrP supplementation. Further correlation analyses revealed positive associations between *Alistipes* and BW, ADG and ADFI, and negative correlations with *HSPs*. By contrast, *Lactobacillus* and *Parabacteroides* were negatively correlated with BW and ADG, and positively correlated with *HSPs*. In conclusion, dietary supplementation with CrP considerably enhances growth performance, promotes intestinal repair and restores homeostasis in heat-stressed broilers.

**Supplementary Information:**

The online version contains supplementary material available at 10.1007/s44154-026-00320-6.

## Introduction

With the intensification of global warming and the continuous development of intensive farming systems, heat stress in broilers has become increasingly prominent, posing severe challenges to the poultry industry (Nawab et al. 2018). Broilers lack sweat glands and are covered with feathers, and their primary heat dissipation mechanism relies on respiratory cooling, which renders them highly sensitive to high ambient temperatures (Khan et al. 2023). When the heat stress index (HSI = Relative Humidity (RH, %) + Fahrenheit (°F)) exceeds 160 and effective is absent ventilation in the poultry house, the flock will experience heat stress (Kim et al. 2025). During heat stress, broilers exhibit open-mouth breathing, wing spreading, and lethargy, along with a significant reduction in feed intake and a marked increase in water consumption (Wang et al. 2018). Chronic heat stress not only induces metabolic disorders and damages visceral organs in broilers but also impairs their production performance and carcass quality, and can even result in massive mortality, leading to substantial economic losses (Oluwagbenga and Fraley 2023). Yellow-feathered broilers, a distinctive indigenous chicken breed in China, are widely raised in southern regions. During summers, the high temperature and humidity in southern China exacerbate heat stress in these birds. As breeding scale expands annually, the development of effective nutritional strategies to mitigate heat stress in yellow-feathered broilers has become increasingly urgent.

Chromium is an essential trace element and a key component of glucose tolerance factor which enhances the binding affinity of insulin to its receptors, thereby promoting glucose uptake and utilization and ultimately improving production performance (Cupo and Donaldson 1987; Brooks et al. 2016). Chromium is widely used in poultry feed supplements, available in both inorganic and organic forms. Among them, organic chromium has a higher bioavailability than inorganic chromium and is more easily absorbed and metabolized (Safwat et al. 2020). Adequate chromium supplementation can promote growth and development in poultry and improve feed utilization efficiency (Zhang et al. 2024; Chen et al. 2025). During heat stress, animals secrete catecholamines to promote the mobilization of chromium, thereby supporting energy metabolism, and depleting tissue reserves and increased excretion, leading to chromium deficiency (Toghyani et al. 2012). Chromium supplementation, in turn, can replenish chromium reserves in the body, significantly alleviate the decline in broiler production performance induced by heat stress, and mitigate oxidative stress (Sahin et al. 2017). Thus, timely supplementation of chromium effectively alleviates heat stress in poultry.

The gut microbiota, often referred to as the body’s ‘second genome’, plays a vital role in host nutrition, metabolism, and immunity (Hu et al. 2025). Heat stress impaired the intestinal structure of broilers and disturbed the homeostasis intestinal microbial, thereby compromising intestinal health (Shaukat et al. 2025). Previous studies have shown that dietary supplementation with 0.4 mg/kg chromium picolinate can improve glucose homeostasis, enhance production performance, and support gut microbial balance in heat-stressed Arbor Acres (AA) broilers (Wang et al. 2022). However, prolonged exposure to high doses of chromium significantly alters the diversity and composition of poultry gut microbiota, and thereby impair intestinal health (Li et al. 2021). Recent studies on organic chromium alleviating heat stress have focused primarily on white-feathered broilers, with limited reports on yellow-feathered broilers. This study aimed to investigate the effects of CrP on production performance and intestinal health in heat-stressed yellow-feathered broilers, to provide a theoretical basis for mitigating heat stress through nutrition regulation.

## Result

### Rectal temperature

The effects of CrP on rectal temperature in heat-stressed yellow-feathered broilers are shown in Figure S2. Compared with the CON group, the HS group showed an increase (*p* < 0.05) in rectal temperature on days 3, 7, 10 and 14. In contrast, the PAC group had lower (*p* < 0.05) rectal temperature than the HS group at the same time points.

### Growth performance

Heat stress decreased (*p* < 0.05) BW, ADG and ADFI, and increased (*p* < 0.05) FCR (Table 1). CrP supplementation reversed these effects, improving (*p* < 0.05) BW and ADG and reducing (*p* < 0.05) FCR compared with the HS group.

**Table 1:** The impact of PAC on growth performance of yellow-feathered broilers under heat stress

Index	CON	HS	PAC
Initial BW (g)	1119.44 ± 24.55	1108.23 ± 36.83	1149.33 ± 48.83
BW (g)	2242.22 ± 37.81a	1916.88 ± 33.13b	2164.60 ± 53.39a
ADG (g)	66.55 ± 1.20a	49.09 ± 0.96c	56.54 ± 2.39b
ADFI (g)	163.56 ± 3.26a	137.38 ± 3.55b	142.20 ± 4.91b
FCR (g/g)	2.46 ± 0.02b	2.810.10 ± 0.10a	2.53 ± 0.05b
Mortality (%)	0.00	13.33	0.00

Data are means ± SD. Each value represents the mean of 6 cages with 10 broilers per cage. Tukey values in rows with different letters differ significantly (*p* < 0.05). *BW* Body weight, *ADG* Average daily gain, *ADFI* Average daily feed intake, *FCR* Feed Conversion Ratio

### Blood parameters and intestinal antioxidant indicators

**Fig. 1:**
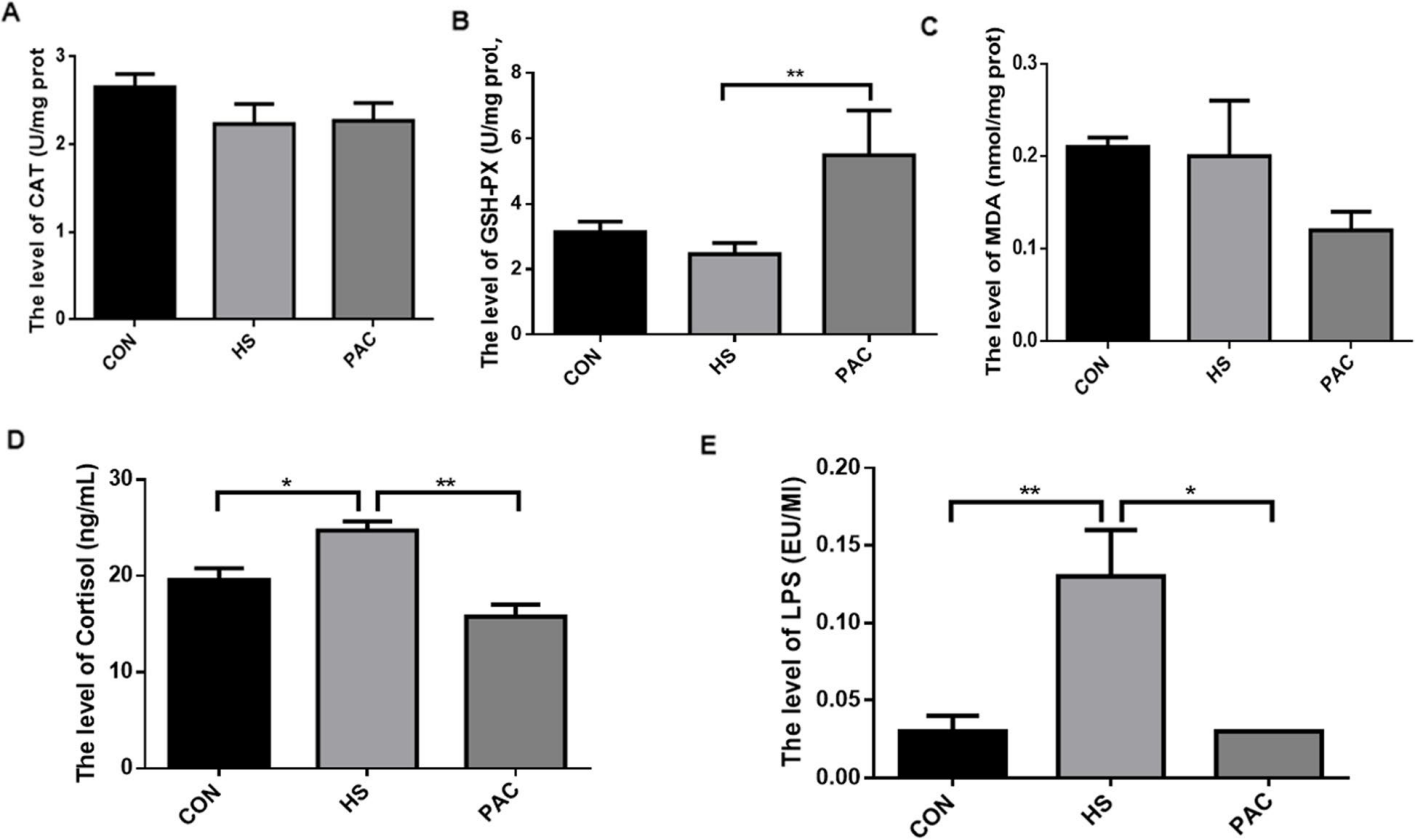
Blood parameters and intestinal antioxidant indicators. **A** The level of CAT; **B** The level of GSH-Px; **C** The level of MDA; **D** The level of Cortisol; **E** The level of LPS. The results were expressed as the mean ± SD; *n* = 6 birds per group and * means *p* < 0.05, ** means *p* < 0.01. CON: Control group, fed with basal diet and maintained room temperature (26 ± 1 °C); HS: Fed with basal diet and performed cycle heat stress; PAC: Fed the basal diet supplemented with 0.2 mg/kg chromium propionate and performed cycle heat stress

The effects of CrP on plasma biochemical parameters and intestinal antioxidant indices are presented in Fig. 1. Heat stress increased (*p* < 0.05) plasma lipopolysaccharide (LPS) and corticosterone (CORT) levels. Conversely, the PAC group had lower (*p* < 0.05) LPS and CORT levels, and higher (*p* < 0.05) GSH-Px activity, compared with the HS group.

### Intestinal morphometry

**Fig. 2:**
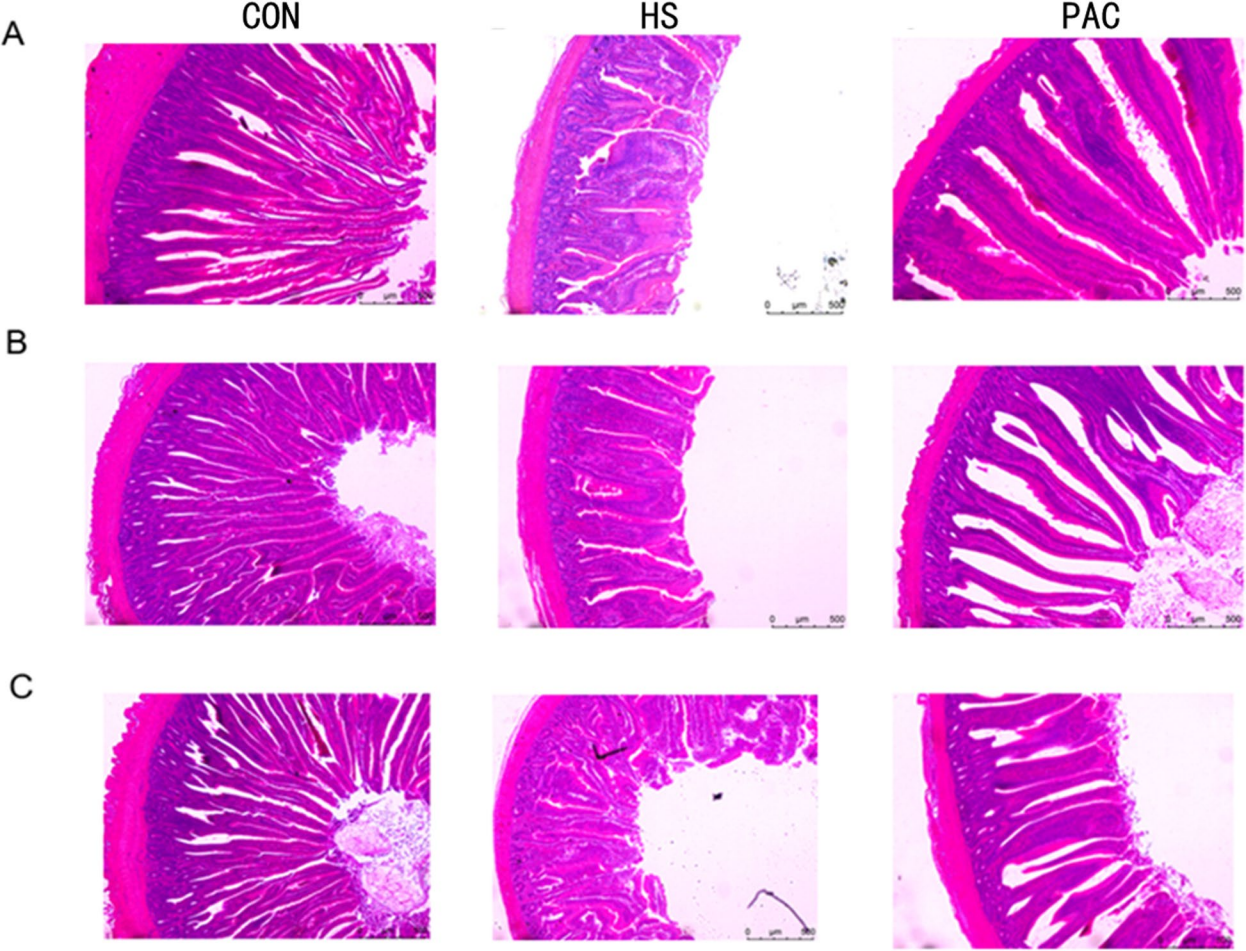
Intestinal morphometry. H&E staining observations of gut tissues of broilers in each group (bar 500 μm). **A** duodenum; **B** jejunum; **C** ileum. HS: Fed with basal diet and performed cycle heat stress; PAC: Fed the basal diet supplemented with 0.2 mg/kg chromium propionate and performed cycle heat stress

Heat stress decreased (*p* < 0.05) the absolute lengths of duodenum, jejunum and ileum (Table 2). CrP supplementation increased (*p* < 0.05) the lengths of jejunum and ileum when compared with the HS group. The effects of CrP on intestinal morphology are shown in Table 3 and Fig. 2. Heat stress decreased (*p* < 0.05) VH in the duodenum and jejunum, increased (*p* < 0.05) CD in all intestinal segments, and decreased (*p* < 0.05) V/C ratio in all intestinal segments. In contrast, CrP supplementation increased (*p* < 0.05) VH and V/C, and decreased (*p* < 0.05) CD, in all intestinal segments compared with the HS group.

**Table 2:** Effects of PAC on intestinal index of yellow-feathered broilers under heat stress

Index	CON	HS	PAC
Duodenum length (cm)	26.83 ± 0.78a	23.42 ± 1.20b	25.38 ± 0.63ab
Jejunum length (cm)	55.42 ± 1.69a	50.17 ± 1.59b	56.25 ± 2.18a
Ileum length (cm)	58.17 ± 2.20a	49.42 ± 1.50b	57.13 ± 1.54a
Duodenum Index (cm/kg)	11.88 ± 0.31	11.79 ± 0.66	11.42 ± 0.29
Jejunum Index (cm/kg)	24.10 ± 0.80	25.20 ± 0.85	24.24 ± 0.99
Ileum Index (cm/kg)	25.23 ± 0.86	24.91 ± 1.01	25.99 ± 0.62

Data are means ± SD. Each value represents the mean of 6 birds for each treatment. Tukey values in rows with different letters differ significantly (*p* < 0.05). The calculate of Index = length/Body weight

**Table 3:** Effects of PAC on intestinal morphology of yellow-feathered broilers under heat stress

Index	CON	HS	PAC
Du VH (μm)	2027.58 ± 121.41a	1616.16 ± 72.36b	1963.12 ± 48.60a
Du CD (μm)	127.35 ± 10.75c	265.58 ± 15.36a	200.25 ± 25.69b
Du V/C	18.05 ± 1.26a	6.87 ± 0.38c	11.19 ± 0.31b
Je VH (μm)	1803.73 ± 70.77a	1236.13 ± 65.09c	1671.38 ± 20.65b
Je CD (μm)	120.54 ± 9.57c	205.41 ± 13.41a	150.20 ± 7.64b
Je V/C	16.06 ± 0.99a	6.20 ± 0.38c	11.06 ± 0.24b
I VH (μm)	1040.61 ± 46.06ab	929.38 ± 68.74b	1098.73 ± 52.63a
I CD (μm)	82.48 ± 5.47b	177.82 ± 11.16a	85.04 ± 2.42b
I V/C	15.26 ± 1.64a	5.27 ± 0.28c	12.85 ± 0.47b

Data are means ± SD. Each value represents the mean of 6 birds for each treatment. Tukey values in rows with different letters differ significantly (*p* < 0.05). *Du* Duodenum, *Je* Jejunum, *I* Ileum, *VH* villus height, *CD* crypt depth, *V/C* VH/CD

### Gene expression

**Fig. 3:**
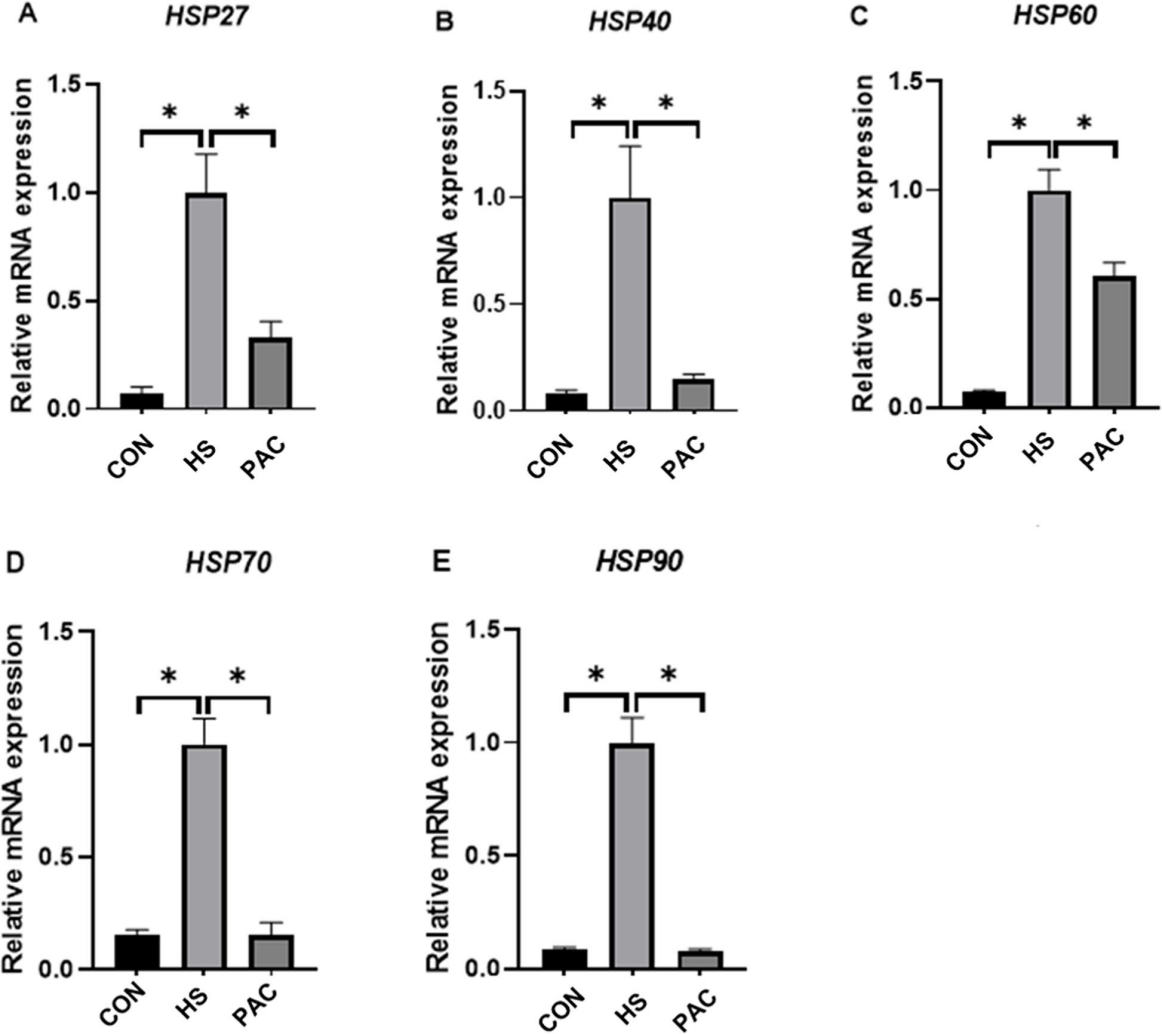
Gene expression of HSPs. The results were expressed as the mean ± SD; *n* = 6 birds per group and * means *p* < 0.05, ** means *p* < 0.01. CON: Control group, fed with basal diet and maintained room temperature (26 ± 1 °C); HS: Fed with basal diet and performed cycle heat stress; PAC: Fed the basal diet supplemented with 0.2 mg/kg chromium propionate and performed cycle heat stress

**Fig. 4:**
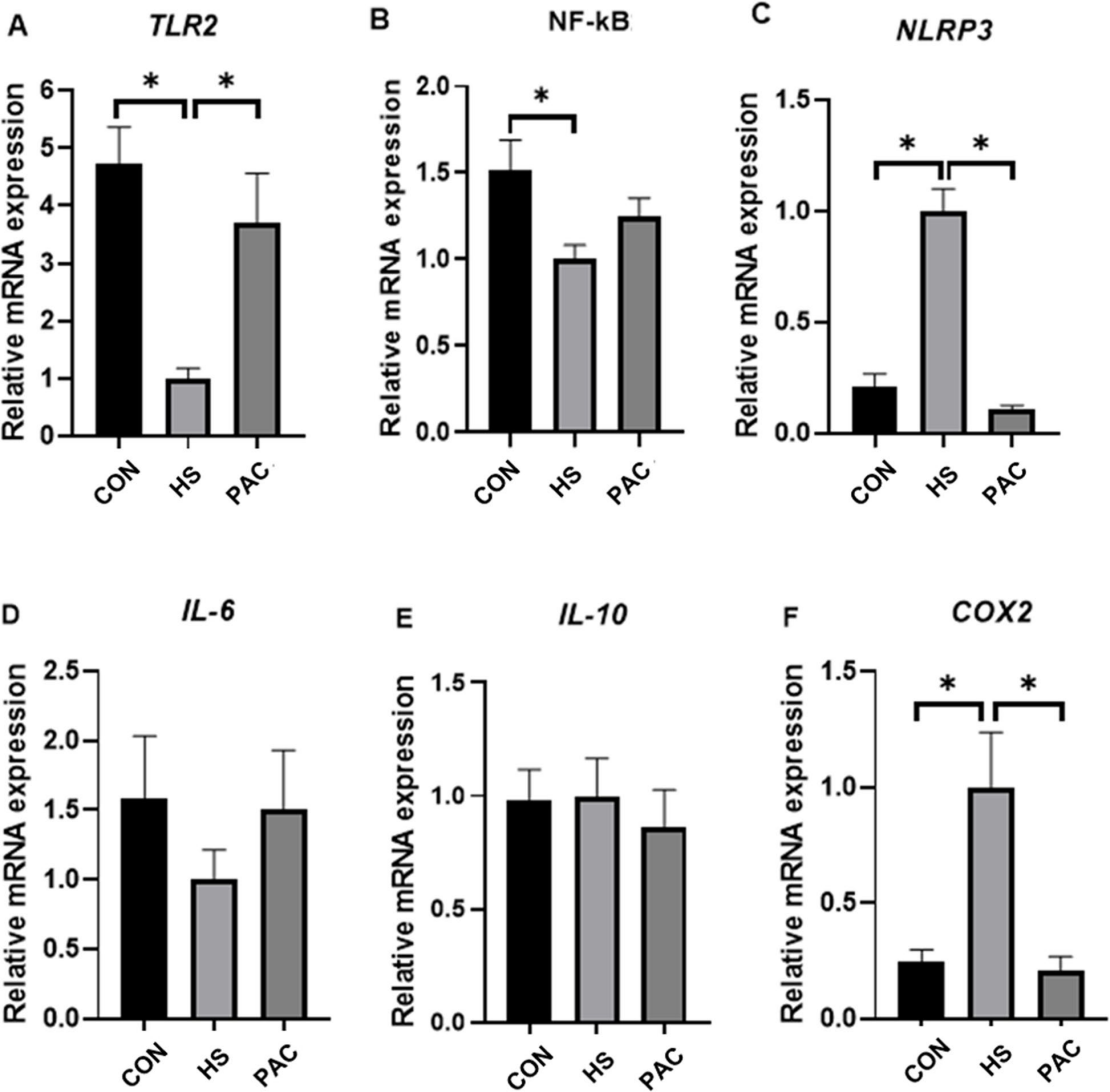
Gene expression of inflammation. The results were expressed as the mean ± SD; *n* = 6 birds per group and * means *p* < 0.05, ** means *p* < 0.01. CON: Control group, fed with basal diet and maintained room temperature (26 ± 1 °C); HS: Fed with basal diet and performed cycle heat stress; PAC: Fed the basal diet supplemented with 0.2 mg/kg chromium propionate and performed cycle heat stress

**Fig. 5:**
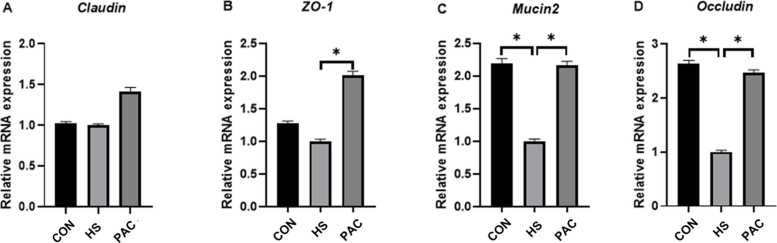
Gene expression of intestinal barrier function. The results were expressed as the mean ± SD; *n* = 6 birds per group and * means *p* < 0.05, ** means *p* < 0.01. CON: Control Group, fed with basal diet and maintained room temperature (26 ± 1 °C); HS: Fed with basal diet and performed cycle heat stress; PAC: Fed the basal diet supplemented with 0.2 mg/kg chromium propionate and performed cycle heat stress

Figure 3 illustrates the effects of CrP on the expression of heat shock protein (*HSPs*) genes in the jejunal mucosa. Heat stress upregulated (*p* < 0.05) the expression of *HSP27*, *HSP60* and *HSP90*. CrP downregulated (*p* < 0.05) *HSP27* and *HSP60* expression when compared with that in the HS group. Regarding inflammation-related genes (Fig. 4), heat stress downregulated (*p* < 0.05) *TLR2* and *NF-κB* expression and upregulated *COX2* and *NLRP3* expression. The PAC group upregulated (*p* < 0.05) *TLR2* and *IFN-γ* expression and downregulated *COX2* and *NLRP3* expression. With regards to intestinal barrier function genes (Fig. 5), heat stress downregulated (*p* < 0.05) *Mucin2* and *Occludin* expression. CrP supplementation upregulated (*p* < 0.05) the expression of *ZO-1*, *Mucin2* and *Occludin*.

### Cecal microbiota composition

**Fig. 6:**
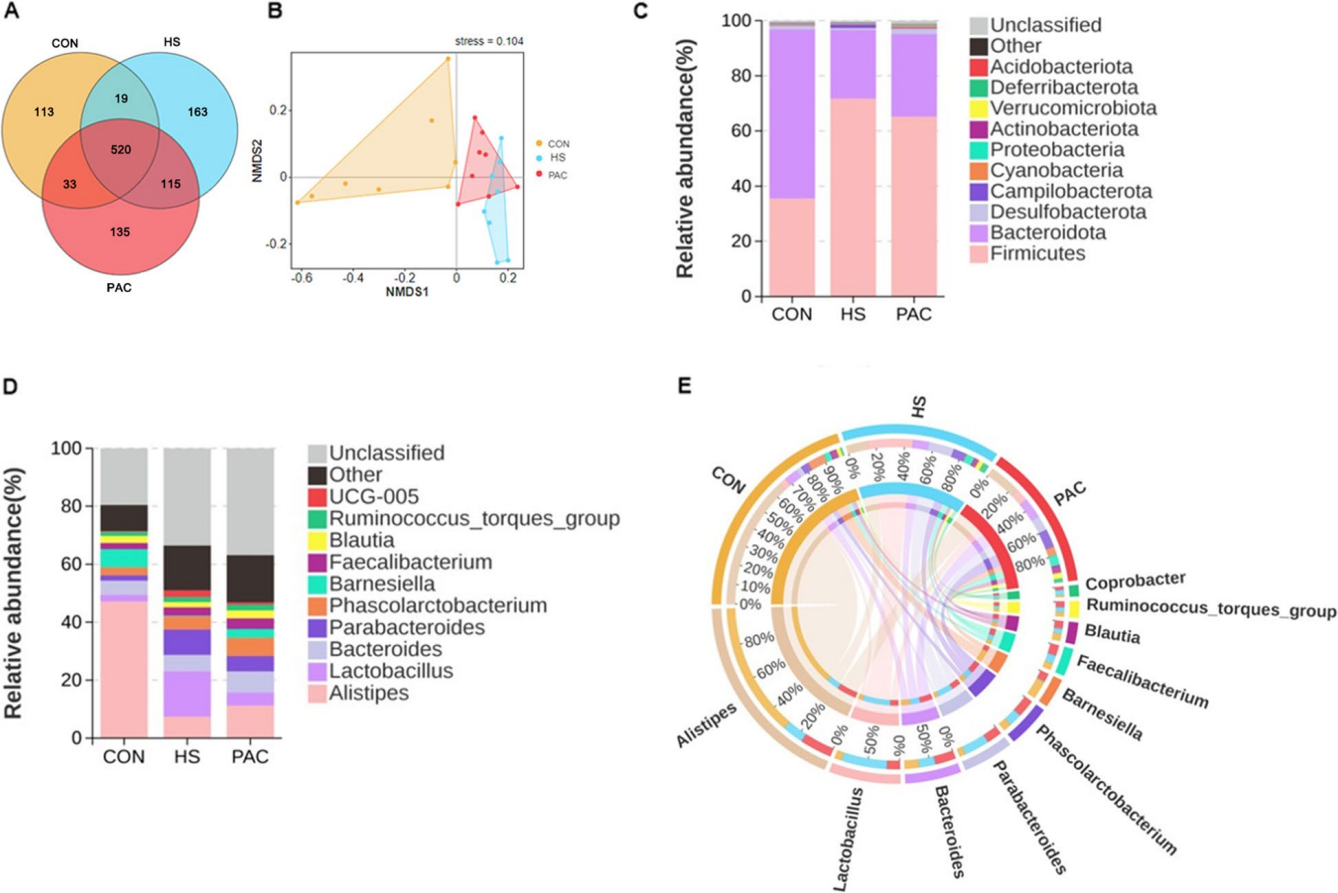
Analysis of cecal microbiota composition. **A** The operational taxonomic unit (OTU) composition of the cecal microbiota; **B** The Non-metric multidimensional scaling (NMDS) analysis; **C** The phylum level relative abundance; **D** The genus level relative abundance; **E** Circos plots. CON: Control group, fed with basal diet and maintained room temperature (26 ± 1 °C); HS: Fed with basal diet and performed cycle heat stress; PAC: Fed the basal diet supplemented with 0.2 mg/kg chromium propionate and performed cycle heat stress

The effects of CrP on the OTU composition are shown in Fig. 6A. The CON, HS, and PAC groups harbored 113, 163, and 135 unique OTUs, respectively. As presented in Table 4 and S3A, heat stress increased (*p* < 0.05) the Sobs, Shannon, Simpson, Chao1, and ACE compared to the CON group, indicating elevated microbial richness and diversity. Non-metric multidimensional scaling (NMDS) analysis (Fig. 6B) revealed distinct clustering patterns among groups: the CON group formed a separate cluster from both HS and PAC groups, while the HS and PAC groups exhibited substantial overlap, suggesting higher microbial similarity between these two groups.

**Table 4:** Effects of PAC on microbial alpha diversity in the cecum of yellow-feathered broilers under heat stress

Index	CON	HS	PAC
Sob	792.63 ± 74.56b	999.13 ± 32.96a	992.00 ± 36.89a
Shannon	4.67 ± 0.56b	6.21 ± 0.20a	6.26 ± 0.14a
Simpson	0.77 ± 0.07b	0.95 ± 0.01a	0.96 ± 0.00a
Chao1	875.10 ± 74.56b	1086.41 ± 30.84a	1069.82 ± 32.89a
ACE	916.05 ± 79.02b	1140.16 ± 32.70a	1120.19 ± 35.02a

Data are means ± SD. Each value represents the mean of 6 birds for each treatment. Tukey values in rows with different letters differ significantly (*p* < 0.05)

**Fig. 7:**
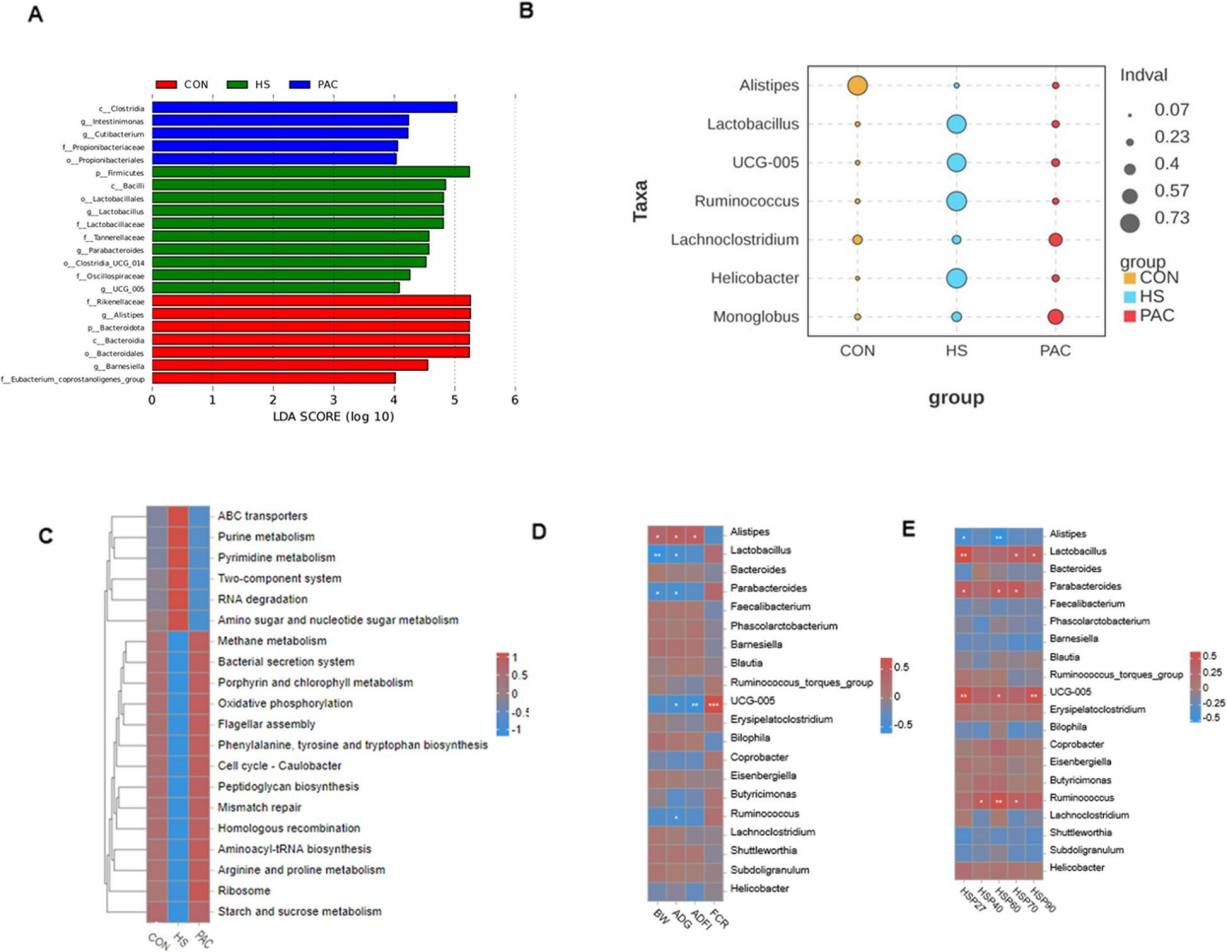
Analysis of cecal microbiota function. **A** LEfSe analysis of differential species for biomarkers on microbiota structure in groups. Different color expresses different group. The abscissa is the LDA score (log10), and the ordinate is the species in different categorical level; **B** Indicator analysis; **C** Functional annotation of the cecal microbiota; **D** The correlation heatmap between gut microbiota and production performs; **E** The correlation heatmap between gut microbiota and HSPs. CON: Control group, fed with basal diet and maintained room temperature (26 ± 1 °C); HS: Fed with basal diet and performed cycle heat stress; PAC: Fed the basal diet supplemented with 0.2 mg/kg chromium propionate and performed cycle heat stress

At the phylum level (Fig. 6C and S3B), heat stress increased (*p* < 0.05) the relative abundance of *Firmicutes* and decreased *Bacteroidetes* compared to the CON group, and PAC decreased the abundance of *Firmicutes* and increased *Bacteroidetes* compared to the HS group. At the genus level (Fig. 6D, Figure S3C), heat stress reduced (*p* < 0.05) the abundance of *Alistipes* and increased *Lactobacillu* compared to the CON group. Conversely, the PAC exhibited lower abundances of *Lactobacillus* and higher *Alistipes* abundance compared to the HS group. Linear discriminant analysis effect size (LEfSe) identified *Lactobacillus* and *Parabacteroides* as the dominant taxa in the HS group, whereas *Intestinimonas* and *Cutibacterium* were enriched in the PAC group (Fig. 7A and B).

Functional annotation of the cecal microbiota (Fig. 7C) revealed 20 major metabolic pathways, including ABC transporters, purine/pyrimidine metabolism, two-component system, and RNA degradation. The CON and PAC groups shared similar functional profiles, which differed significantly from those of the HS group. Heat stress was associated with reduced carbohydrate and amino acid metabolism and increased RNA degradation, whereas chromium propionate supplementation reversed these effects, suggesting mitigation of heat stress-induced metabolic disturbances.

The correlation analysis between cecal microbial genera and growth performance/*HSPs* is presented in Fig. 7D and E. *Alistipes* exhibited positive correlations (*p* < 0.05) with BW, ADG, and ADFI, while showing a negative correlation (*p* < 0.05) with the expression levels of *HSP27* and *HSP60*. In contrast, *Lactobacillus* and *Parabacteroides* showed a negative correlation (*p* < 0.05) with BW and ADG but positively correlated with the expression of *HSP27, HSP70*, and *HSP90*. *UCG-005* showed a negative correlation (*p* < 0.05) with ADG and ADFI but positively correlated with FCR and the expression of *HSP27, HSP60,* and *HSP90*.

## Discussion

**Fig. 8:**
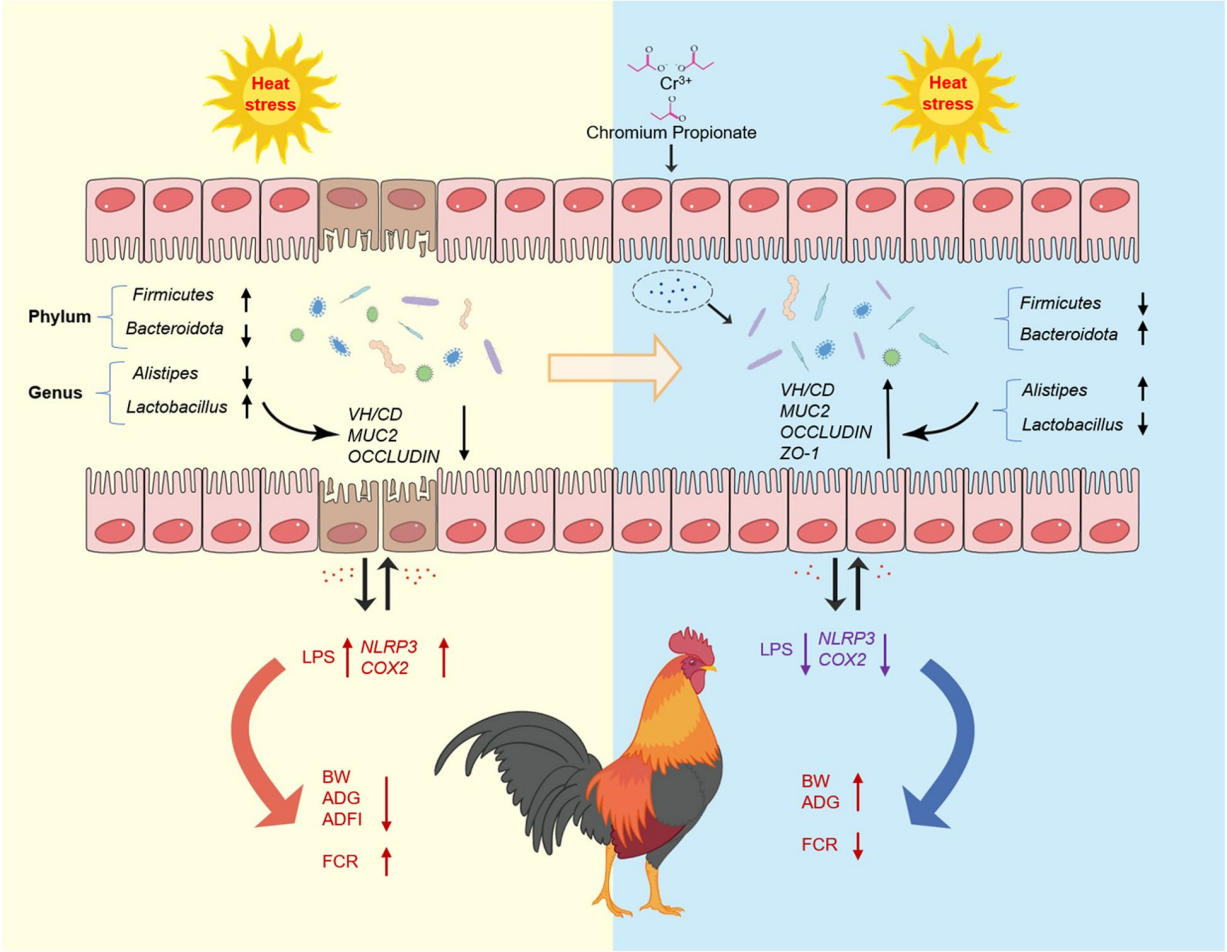
Schematic diagram of regulatory network of chromium propionate alleviates the damage caused by heat stress by improving the imbalance of intestinal flora

Growth retardation in broilers under heat stress is primarily attributed to a sharp reduction in feed intake. Moreover, heat stress impairs growth performance by disrupting systemic metabolism and reducing nutrient utilization efficiency (Lara and Rostagno 2013; Sumanu et al. 2022). Hatipoglu et al. reported that chronic heat stress (5–7 h/day at 34–36 °C for 42 days) significantly decreased BW and increased FCR in Ross 308 broilers (Hatipoglu et al. 2024). Won et al. found that 14days of cyclic heat stress (8 h/day at 32 °C) significantly reduced BW and ADG, and increased FCR in Ross 308 broilers (Won et al. 2023). Our results demonstrate that dietary supplementation with CrP significantly improved production performance of broiler during heat stress. Jahanian et al. reported that dietary supplementation with 500 or 1000 ppb chromium methionine could increase feed intake and body weight as well as improve feed efficiency in Ross 308 broilers under heat stress conditions (Jahanian and Rasouli 2015). The growth-promoting effect of chromium may be associated with its regulatory role in glucose metabolism. A previous study found that Cr (III) binds to ATP synthase and inhibits its activity, thereby activating the AMPK pathway and improving glucose metabolism (Wang et al. 2023). Notably, our study also found that CrP supplementation significantly reduced heat stress-induced mortality in broilers. Heat stress activates the HPA axis, leading to excessive CORT secretion that in turn induces systemic metabolic disorders, which is a key cause of broiler mortality (Li et al. 2023). Chromium attenuates HPA axis activation and CORT secretion by improving glucose homeostasis, alleviating metabolic disorders and reducing body temperature, thereby lowering broiler mortality (Sahin et al. 2002). The decreased rectal temperature and CORT levels in the PAC group also confirms the aforementioned viewpoint. These results are consistent with the abovementioned reports, confirming that CrP can effectively alleviate heat stress-induced growth performance decline in yellow-feathered broilers. The underlying mechanism may be related to CrP-mediated improvement of systemic metabolic disorders and promote digestion and absorption rather than increased feed intake (Fig. 8).

The intestine is the primary digestive and absorptive organ in poultry, and its function is severely impaired by heat stress, leading to reduced growth performance (Rostagno 2020). Our study found that the inhibition of growth performance was accompanied by a reduction in the absolute length of the broiler intestine, which further led to a decrease in its nutrient absorption capacity. Additionally, CrP alleviated heat stress-induced intestinal mucosal dysfunction. Song et al. reported that cyclic heat stress impaired intestinal barrier function in AA broilers, characterized by decreased VH and V/C, reduced antioxidant enzyme activities (GSH-Px, SOD, CAT), and downregulated expression of mucosal barrier-related genes (*Claudin-1* and *Muc2*), ultimately leading to a significant reduction in nutrient absorption (Song et al. 2019). Hayat et al. found that 0.10–0.30 mg/kg CrP supplementation significantly increased VH and the V/C ratio while decreased CD in broilers (Hayat et al. 2020). Huang et al. reported that high-temperature damaged broiler intestinal morphology, and dietary CrP supplementation showed a quadratic relationship with duodenal VH and the V/C ratio (Huang et al. 2020). Consistent with these findings, our results also showed an increasing in V/C ratio, GSH-Px activity and intestinal barrier-related gene expression in heat-stressed yellow-feathered broilers. The intestinal protective effect of chromium is mainly attributed to its antioxidant activity. A previous study reported that Cr reduces intracellular ROS levels to counteract iron-induced glucose metabolic disorders (Fan et al. 2024). Another study also indicated that dietary supplementation with 2 mg/kg organic chromium significantly improved the growth performance and antioxidant capacity of Cobb broilers under cyclic heat stress conditions (Rao et al. 2016). In contrast, hexavalent chromium exerts toxic effects by inducing oxidative damage, downregulating intestinal barrier gene expression, such as *ZO-1, Occludin,* and *Mucin2* in poultry (Xing et al. 2022). Furthermore, heat stress increased LPS level, which were alleviated by CrP supplementation. The HS group demonstrated significantly increased gene expressions of *COX2* and *NLRP3* in the jejunal mucosa, which were suppressed by CrP supplementation. These findings suggested that CrP mitigates LPS-induced inflammation via restoring intestinal barrier integrity. Numerous studies have demonstrated that heat stress triggers the inflammatory response in broilers through two distinct pathways: activating the TLR4-NF-κB pathway by promoting LPS release, and activating the NLRP3 inflammasome by increasing intracellular ROS levels, thereby promoting the release of pro-inflammatory cytokines such as interleukin-6 (IL-6) and interleukin-1β (IL-1β), thereby further causing tissue damage (Fan et al. 2020; Mckay et al. 2021; Liu et al. 2022; Du et al. 2024). Hamidi et al. found that heat stress notably increased *IFN-γ* expression, while CrP supplementation decreased *IFN-γ* and increased *NLRP3* gene in broilers (Hamidi et al. 2022). Fraz et al. reported that dietary supplementation with organic chromium considerably downregulated *COX2* expression and upregulated *TLR2* and *IFN-γ* expression (Fraz et al. 2023). Collectively, these findings demonstrate that CrP alleviated heat stress-induced intestinal oxidative damage, preserves intestinal barrier integrity and inhibits inflammation, thereby maintaining normal nutrient digestion and absorption in broilers.

The caecum of poultry harbours a rich and diverse microbial community that plays a pivotal role in feed digestion, nutrient absorption and intestinal development (Mazanko et al. 2022). Previous studies have shown that heat stress impairs the digestive and absorptive capacity of broilers, leading to excess nutrients entering the cecum that are metabolized by pathogenic bacteria, disrupting intestinal microbial homeostasis (Zhang et al. 2023; He et al. 2024). This study revealed that the microbial richness and diversity were higher in the HS group than in the CON and PAC groups. This increase was likely driven by pathogenic bacterial proliferation rather than beneficial microbes. Herein, at the genus level, the HS group showed a significant decrease in *Alistipes* abundance, while CrP supplementation reversed this trend. *Alistipes*, a core anaerobic Gram-negative genus in *Bacteroidota*, is abundant in broiler hindgut (cecum and colon), has been reported to promote the growth of broilers (Torok et al. 2011). Mckenna et al. also reported that *Alistipes* is positively correlated with broiler growth performance and directly linked to glucose metabolism, amino acid metabolism, and nucleotide metabolism (Mckenna et al. 2020). Changes in *Alistipes* abundance are accompanied by the remodeling of amino acid and glucose metabolism. Meanwhile, correlation analysis further confirmed the positive correlation *Alistipes* and broiler production performance, consistent with previous reports. Heat stress impairs intestinal mucosal barrier function and disrupts *Alistipes* homeostasis, making this genus a potential target for CrP-mediated microbial regulation. Goel et al. reported that heat stress reduced cecal *Alistipes* abundance and impaired broiler growth performance (Goel et al. 2021). Another study also found that long-term high-temperature exposure decreased *Alistipes* abundance and impaired immune function in broilers. (Yang et al. 2021). The regulatory effect of *Alistipes* on heat stress may be related to its anti-inflammatory activity. Study showed a negative correlation between *Alistipes* and IBD, and its strains can suppress proinflammatory cytokines, like IL-1β, IL-6 and TNF-α (Older et al. 2025). Another study has also shown that tea polysaccharides ameliorated heat stress-induced intestinal microbial disturbance and inflammation in cattle by increasing *Alistipes* relative abundance (Li et al. 2024). Additionally, *Alistipes* was negatively correlated with *HSP27* and *HSP60* expression, which were downregulated by CrP. HSPs are a class of highly conserved proteins that maintain cellular homeostasis under heat stress (Biswas et al. 2024). Madkour et al. found that 32°C± 2 °C heat stress significantly upregulated *HSP70* and *HSP90* expression (Madkour et al. 2024). Oloruntola et al. reported that under heat stress conditions, Cobb broilers demonstrate a significant increase in caecal microbiota abundance and *HSPs* gene expression, accompanied by a marked decline in growth performance (Oloruntola et al. 2023). Notably, the present study found that the relative abundance of *Lactobacillus* (commonly regarded as a probiotic) in the intestines of broilers increased significantly under heat stress conditions, which negatively correlated with broiler production performance. This might be due to the change in intestinal pH caused by excessive accumulation of lactic acid as a result of heat stress, which induces the over proliferation of *Lactobacillus*, leads to the dysregulation of microbial homeostasis, and further exacerbates intestinal damage (Dean 2007). In summary, CrP alleviates heat stress-induced growth impairment in yellow-feathered broilers by restoring intestinal microbial homeostasis, particularly by increasing *Alistipes* abundance.

## Conclusion

In conclusion, cyclic heat stress reduces growth performance, impairs intestinal barrier function and disrupts microbial homeostasis in yellow-feathered broilers. In contrast, dietary supplementation with 0.2 mg/kg CrP improves growth performance without increasing feed intake, enhances intestinal antioxidant capacity, suppresses inflammatory responses, repairs intestinal barrier integrity and restores cecal microbial balance, thereby mitigating the adverse effects of heat stress. These findings provide experimental evidence for the application of CrP as a nutritional intervention to improve health and production efficiency in heat-stressed poultry.

## Materials and methods

### Experimental materials

A total of 180 35-day-old yellow-feathered broilers were purchased from Jiangsu Lihua Animal Husbandry Co., Ltd., Chromium propionate (with organic chromium content ranging from 0.09% to 0.10%) was purchased from Jiangsu Yishi Biotechnology Co., Ltd.

### Experimental design

A total of 180 35-day-old yellow-feathered broilers with similar initial body weights were randomly divided into 3 groups (6 replicates/group, 10 broilers/replicate). The control group (CON) and the heat stress group (HS) were fed a basal diet, while the heat stress + chromium propionate group (PAC) received the basal diet supplemented with 0.2 mg/kg CrP (calculated based on the organic chromium content). The total experimental period was 21 days, including a 1-week pre-test period with the same CrP supplementation ratio as the formal trial. After 7 days of pre-feeding in all three groups, the HS group and the PAC group were subjected to cyclic heat stress treatment. The program was set as follows: 00:00–06:30 and 22:30–00:00 at 30°C ± 1 °C, totaling 8 h; 06:30–10:30 and 18:30–22:30 at 32°C ± 1 °C, totaling 8 h; 10:30–18:30 at 34°C ± 1 °C, totaling 8 h (detailed in Supplementary Figure S1). The CON group was maintained at 26°C ± 1 °C for 2 weeks.

### Feeding management

The experiment was conducted in an animal nutrition and metabolism climate-controlled chamber (Beijing Kulan Technology Co., Ltd.). Broilers were reared in three-tier cages (40 cm ×45 cm ×45 cm). All broilers had ad libitum access to feed and water; relative humidity was maintained at 60% ± 5%; feces were removed daily; and 24-h lighting was provided. Feed intake, body weight, rectal temperature, etc., were recorded during the period. The diet was in the form of pellets, and its composition and nutritional levels are shown in Table 5. Dietary formulation followed the requirements recommended by the NRC (1994) and the Nutrient Requirements of Yellow-Feathered Broilers (NY/T 3645—2020). All experimental procedures in our study had been were approved by the Animal Ethics Committee of Jiangsu Institute of Poultry Science, China. Surgical and sampling procedures also complied with the “Guidelines on the Ethical Treatment of Experimental Animals” (2006) No. 398 published by the Ministry of Science and Technology, China, and with the “Regulations Regarding the Management and Treatment of Experimental Animals” (2008) No. 45, published by the Jiangsu Provincial People's Government.

**Table 5:** Composition and nutrient levels of diets (air-dry basis) %

Items	Content
Ingredients	
Corn (CP = 7.2%)	69.37
Soybean meal (CP = 43%)	20.00
Corn gluten meal (CP = 60%)	5.00
Soybean oil	3.03
Limestone	0.96
CaHPO_4_	0.52
98% DL-Met	0.15
98.5% Lys	0.29
98% Thr	0.02
98.5% Arg	0.01
Phytase	0.02
Choline chloride	0.10
Trace mineral premix ^1)^	0.20
Vitamin premix ^2)^	0.03
NaCl	0.30
Total	100.00
Nutrient levels ^3)^	
CP	17.00
EE	5.66
CF	2.18
CA	3.80
ME (MJ/kg)	13.18
Ca	0.55
TP	0.42
AP	0.22
Dlys	0.87
DMet/lysP	0.48
DThr/LysP	0.64
DArg/Lysp	1
DTrp/lysp	0.16
DlIe/Lysp	0.79
DVal/Lysp	0.88

^1^Trace mineral premix provided the following per kg of the diet: Mn (MnSO_4_.H_2_O, 32.49% Mn) 60 mg, I (KI, 58% I) 0.35 mg, Fe (FeSO_4_.7H_2_O, 20.09% Fe) 25 mg, Cu (CuSO_4_.5H_2_O) 8 mg, Zn (ZnO, 80.35% Zn) 50 mg

^2^Vitamin premix provided the following per kg of the diet: Vitamin A (retinol) 4000 IU, Vitamin D3 (cholecalciferol) 1600 IU, Vitamin E (tocopheryl acetate), 18 mg Vitamin K3 1.5 mg, Vitamin B1 1.0 mg, Vitamin B2 3.0 mg, Vitamin B6 3.0 mg, Vitamin B12 0.005 mg, pantothenic acid 8.0 mg, nicotinic acid 20 mg, folic acid 20 mg

^3^*CP* Crude Protein, *EE* Ether Extract, *CF* Crude Fiber, *CA* Crude Ash, *ME* Metabolizable Energy, *TP* Total Phosphorus, *AP* Available Phosphorus

^4^ME and AP were calculated values, referred to NY/T3645-2020; CP, Ca and TP were measured values, referred to GB/T 6432–2018, GB/T 6436–2018, GB/T 6437–2018, respectively

### Detection of production performance

Growth performance was recorded throughout the experiment. Broilers were fasted for 12 h before weighing, and body weight was measured per replicate at 35 and 56 days of age (7:00 AM). At the end of the experiment, residual feed was weighed to calculate the average daily weight gain (ADG), average daily feed intake (ADFI), feed conversion ratio (FCR), and mortality rate during the experimental period.

### Detection of rectal temperature

On days 1, 3, 7, 10 and 14 of the formal trial, rectal temperature was measured for all broilers at 14:00 p.m. The differences in rectal temperatures among groups at different time periods were compared.

### Sample collection and processing

On the 21 st day, tissues and serum samples from 6 birds per treatment group were collected, which randomly selected based on the average body weight of the replicate. The body weight was measured, 4 mL blood was collected using anticoagulant tubes, and plasma was separated by centrifugation at 3000 r/min for 15 min, then stored at −20 °C. All broilers were then euthanized, and tissue samples were collected for subsequent analysis.

### Detection of blood indicators

The plasma CORT content was detected using the QuicKey Pro Chicken CORT ELISA Kit (Elabsciences®, Wuhan, E-OSEL-Ch0002). ELISA procedures were performed strictly according to the manufacturer’s instructions, briefly as follows: kit components were equilibrated at room temperature (25 ± 2 °C) for 30 min; wash buffer was diluted at the specified ratio; the standard was reconstituted and subjected to serial two-fold dilution, with blank control wells set up. Each well was loaded with 50 μL standard or sample (triplicate), followed by 50 μL HRP-conjugated secondary antibody (excluding blank wells). Plates were sealed and incubated at 37 °C for 60 min, then washed five times and patted dry completely. Then, 90 μL substrate solution was added per well, and plates were incubated at 37 °C for 15 min in the dark, followed by 50 μL stop solution to terminate the reaction. The optical density (OD) values were immediately measured at 450 nm using a microplate reader, and the standard curve was plotted to calculate the CORT concentrations in the samples. The plasma endotoxin was detected using the Endotoxin Detection Kit (Xiamen Bioendo Technology Co., Ltd, MKC0505). The specific operation methods were carried out in accordance with the instructions of the relevant kits.

### Morphological analysis

A 1-cm segment of the mid-jejunum was collected, fixed in 4% paraformaldehyde, and embedded in paraffin for sectioning and hematoxylin–eosin (H&E) staining. For each section, villus height (VH) and crypt depth (CD) in each group were measured using image analysis software (specify the software if applicable, e.g., Image-Pro Plus). One section was prepared per bird, and the VH and CD of 10 villi were measured for each section. The mean values were calculated and used as the villus height and crypt depth for the individual sample, which were then subjected to subsequent statistical analyses. The villus height/crypt depth ratio (V/C) was calculated to evaluate jejunal mucosal morphological changes.

### Determination of antioxidant indicators

The activities of catalase (CAT), glutathione peroxidase (GSH-Px), and the content of malondialdehyde (MDA) in the jejunal mucosa were measured using commercial kits (Nanjing Jiancheng Bioengineering Institute, Nanjing, China).

### RNA extraction and quantitative real-time PCR

Total RNA was extracted from the jejunal mucosa using the RNA-easy Isolation Reagent (Vazyme, catalog no. R701-02-AA) following the manufacturer’s instructions. RNA purity and concentration were assessed using a NanoDrop 2000 spectrophotometer (Thermo Fisher Scientific, Waltham, MA, USA) by measuring absorbance at 260/280 nm, and samples were diluted to a uniform concentration. Reverse transcription-quantitative polymerase chain reaction (RT-qPCR) was performed using the RT-qPCR kit (Novoprotein, catalog no. E301-01A). cDNA stored at −20 °C. Quantitative real-time PCR (qPCR) was conducted using Hieff® qPCR SYBR Green Master Mix (Yeasen, Shanghai, China) on a StepOnePlus Real-Time PCR System (Thermo Fisher Scientific, Waltham, MA, USA). Each sample was analyzed in duplicate under the following cycling conditions: initial denaturation at 95 °C for 5 min, followed by 40 cycles of 95 °C for 10 s and 65 °C for 30 s. Gene-specific primers were synthesized by Suzhou Genewiz Biotechnology Co., Ltd., and their sequences are listed in Table 6. The qPCR reactions were conducted on an Applied Biosystems Step One Plus Real-Time PCR System, and the relative gene expression levels were calculated using the 2^−ΔΔCt^ method.

**Table 6:** Primer information

Gnens	Primer sequences (5’−3’)	Product (bp)	Temperature (°C)	Genebank Number
*GAPDH*	F: GCCCAGAACATCATCCCA	137	60	NM_204305.2
	R: CGGCAGGTCAGGTCAACA			
*HSP27*	F: GGTGGTGAAGACTAAGGATAA	209	54	NM_205290.2
	R: TCTCGGATGACTGGATGG			
*HSP40*	F: CCACCAGTTATCCGTAAGTT	101	55	XM_046935213.1
	R: CACCACGACCTTCACATT			
*HSP60*	F: TTGATGGAGAAGCCCTCAGC	151	60	NM_001012916.3
	R: TCCTCTCCAAACACAGCACC			
*HSP70*	F: GTGTCCATCCTTACCATTGA	128	55	NM_001006685.2
	R: TGCTTACGCTTGAACTCTT			
*HSP90*	F: CACAGTCCAAGTCCATAGG	110	54	XM_040672816.2
	R: CATCTCTTGAGTAACGAACAC			
*Claudin*	F: CTGATTGCTTCCAACCAG	140	56	NM_001013611.2
	R: CAGGTCAAACAGAGGTACAAG			
*ZO-1*	F: CTTCAGGTGTTTCTCTTCCTCCTC	131	60	XM_046925214.1
	R: CTGTGGTTTCATGGCTGGATC			
*Mucin2*	F: GTGAAGACCCTGATGAAA	219	55	NM_001318434.1
	R: GTGAACACTGGCGAGAAT			
*Occludin*	F: TCATCGCCTCCATCGTCTAC	240	60	XM_046904540.1
	R: TCTTACTGCGCGTCTTCTGG			
*TLR2*	F: CCACAGTTCTCCATCATCA	106	55	XM_046915414.1
	R: TCAGACTTCCAGGCTCAT			
	R: CATTGTCTCCATTGTCATCC			
*NF-κB*	F: AACTTCACTCTGGCACTAC	173	55	XM_046919840.1
	R: CGCACCTCAATGTCATCT			
*IL-6*	F: CCTCCTCGCCAATCTGAA	100	56	NM_204628.2
	R: CCTCACGGTCTTCTCCATA			
*IL-10*	F: CTGTCACCGCTTCTTCAC	162	55	NM_001004414.4
	R: CACTTCCTCCTCCTCATCA			
*COX2*	F: GGAGTCTGGAACATCATCA	167	54	XM_046922435.1
	R: AGGCTTCTTGTGTAATAGGA			
*NLRP3*	F: CACCAACCTGACCATAGC	191	55	XM_046918112.1
	R: ACTCCATCATCACCTTCCT			

###  16 s RNA sequencing and analysis

Genomic DNA from cecal content samples was extracted using the Fecal Genomic DNA Extraction Kit (Tiangen Biotech Co., Ltd., Beijing, China; Cat. No. DP328-02). Subsequently, DNA purity and concentration were determined by 1% agarose gel electrophoresis, and the DNA was diluted to 1 ng/μL to serve as the amplification template. The V4 hypervariable regions of the bacterial 16S rRNA gene were amplified by using specific primers: 515F (5’-GTTTCGGTG CCAGCMGCCGCGGTAA-3’) and 806R (5’-GCCAATGGACTACHVGGGTWTCTAAT-3’). Library construction was performed using the NEB Next^➅^ Ultra™ II FS DNA PCR-Free Library Prep Kit (New England Biolabs, Ipswich, MA, USA). The constructed libraries were quantified using Qubit and qPCR. After passing quality control, sequencing was performed on an Illuminaon NovaSeq 6000 platform with paired-end 250 bp reads (Illumina Inc., San Diego, CA; OE Biotech Company, Shanghai, China.)

Reads from each sample were concatenated using FLASH (Version 1.2.11), and the concatenated sequences were designated as Raw Tags. The concatenated Raw Tags were subjected to strict filtering using FASTP (Version 0.23.1) to obtain Clean Tags. Usearch (v11.0.667) clustered clean tags into OTUs (97% similarity) via UPARSE. Chimeras were removed using UCHIME. Effective tags were used for OTU abundance statistics. Representative sequences were annotated using SILVA (v138.2) via RDP classifier. Sample complexity (Alpha Diversity) and multi-sample comparative (Beta Diversity) analyses were used to investigate the diversity of the samples. Sample complexity was analyzed using Tukey's test. The multi-sample comparative analysis was performed using non-metric multidimensional calibration (NMDS). LEfSe analysis was conducted with the threshold as follow: linear discriminant analysis (LDA) > 4 and *p* < 0.05. The potential function of the microbial communities was predicted using the Tax4Fun tool based on the 16S rRNA gene sequencing data, which annotated to the KEGG database. The functional differences were visualized using STAMP software.

### Statistical analysis

The experiment adopted a randomized complete block design (RCBD), with the cage serving as the experimental unit to avoid cage effects. Experimental data were recorded and processed using Microsoft Excel 2013 (Microsoft Corporation, Redmond, WA, USA). Statistical analyses were performed using SPSS software version 20.0 (IBM Corporation, Armonk, NY, USA) with one-way ANOVA followed by Tukey's HSD post-test for multiple comparisons. Results are presented as mean ± standard deviation, and a *p*-value < 0.05 was considered statistically significant.

## Supplementary Information

Supplementary Material 1: S1. Program setting of cyclic heat stress. 

Supplementary Material 2: S2. Rectal temperature. 

Supplementary Material 3: S3 A. Effects of CrP on microbial alpha diversity in the cecum of yellow-feathered broilers under heat stress; B The relative abundance in Phylum Level; C The relative abundance in Genus Level. 

## Data Availability

The data analyzed during the current study are available from the corresponding author on reasonable request. The 16S rRNA sequencing data can be found in NCBI’s Sequence Read Archive (SRA) database under accession number PRJNA1308318.

## Abbreviations

ADFI: Average daily feed intake
ADG: Average daily gain
BW: Body weight
CAT: Catalase
CD: Crypt depth
COX2: Cyclooxygenase-2
Cr: Chromium
FCR: Feed conversion ratio
GSH-Px: Glutathione peroxidase
GTF: Glucose tolerance factor
HSPs: Heat shock proteins
IL-6: Interleukin-6
IL-10: Interleukin-10
NLRP3: NOD-, LRR- and pyrin domain-containing protein 3
MDA: Malonaldehyde
MUC2: Mucin 2
OUT: Operational taxonomic units
PAC: Propionic acid chromium
PCA: Principal component analysis
PCoA: Principal co-ordinates analysis
qPCR: Quantitative real-time PCR
SOD: Superoxide dismutase
TNFα: Tumor necrosis factor α
VH: Villus height
V/C: Villus height/crypt depth ratio
ZO-1: Zonula occludens-1
