# Supplementary figures and images for: Chromium propionate enhanced the production performance of yellow-feathered broilers under chronic heat stress exposure by improving intestinal health
Source: Stress Biol. 2026 Jun 22;6(1):45. doi: 10.1007/s44154-026-00320-6 (PMC13287288; doi:10.1007/s44154-026-00320-6)

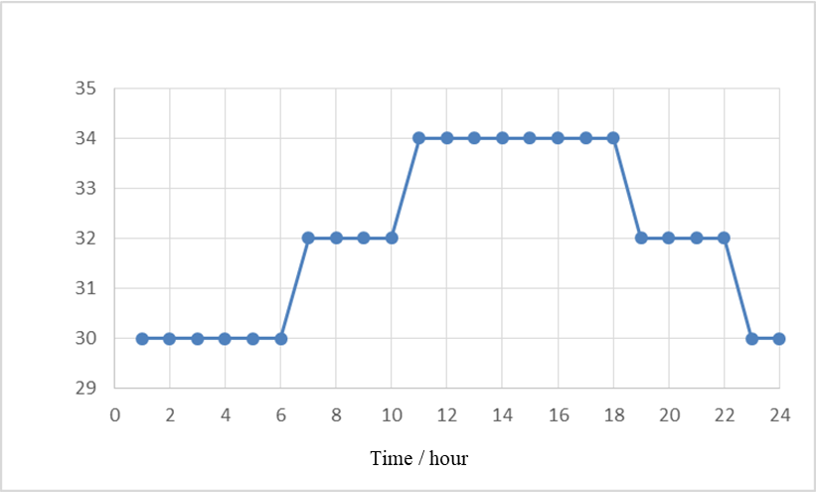

Supplement: Supplementary file 1 — Supplementary Material 1: S1. Program setting of cyclic heat stress. [file 44154_2026_320_MOESM1_ESM.tif]

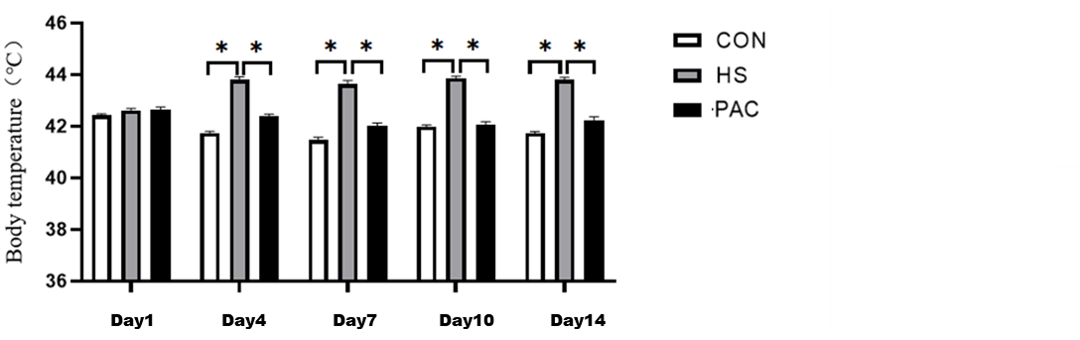

Supplement: Supplementary file 2 — Supplementary Material 2: S2. Rectal temperature. [file 44154_2026_320_MOESM2_ESM.tif]

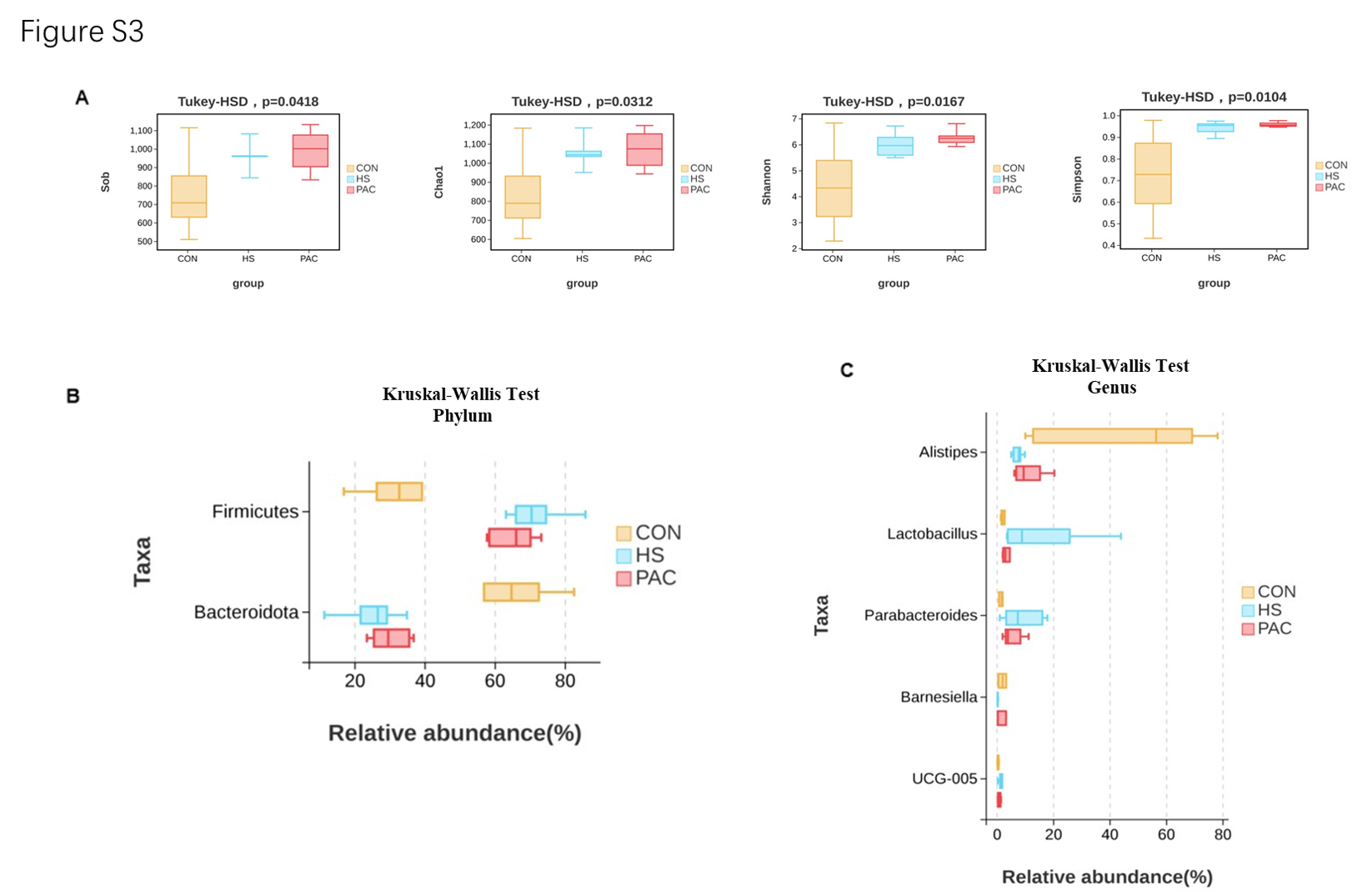

Supplement: Supplementary file 3 — Supplementary Material 3: S3 A. Effects of CrP on microbial alpha diversity in the cecum of yellow-feathered broilers under heat stress; B The relative abundance in Phylum Level; C The relative abundance in Genus Level. [file 44154_2026_320_MOESM3_ESM.tif]
